# Bidirectional regulation of the cGAS-STING pathway in the immunosuppressive tumor microenvironment and its association with immunotherapy

**DOI:** 10.3389/fimmu.2024.1470468

**Published:** 2024-10-11

**Authors:** Yurui Zhang, Yudi Wang, Peizheng Mu, Xiao Zhu, Yucui Dong

**Affiliations:** ^1^ Department of Immunology, Binzhou Medical University, Yantai, China; ^2^ School of Computer and Control Engineering, Yantai University, Yantai, China

**Keywords:** cGAS-STING, tumor, TME, immunotherapy, immune escape

## Abstract

Adaptive anti-tumor immunity is currently dependent on the natural immune system of the body. The emergence of tumor immunotherapy has improved prognosis and prolonged the survival cycle of patients. Current mainstream immunotherapies, including immune checkpoint blockade, chimeric antigen receptor T-cell immunotherapy, and monoclonal antibody therapy, are linked to natural immunity. The cGAS-STING pathway is an important natural immunity signaling pathway that plays an important role in fighting against the invasion of foreign pathogens and maintaining the homeostasis of the organism. Increasing evidence suggests that the cGAS-STING pathway plays a key role in tumor immunity, and the combination of STING-related agonists can significantly enhance the efficacy of immunotherapy and reduce the emergence of immunotherapeutic resistance. However, the cGAS-STING pathway is a double-edged sword, and its activation can enhance anti-tumor immunity and immunosuppression. Immunosuppressive cells, including M2 macrophages, MDSC, and regulatory T cells, in the tumor microenvironment play a crucial role in tumor escape, thereby affecting the immunotherapy effect. The cGAS-STING signaling pathway can bi-directionally regulate this group of immunosuppressive cells, and targeting this pathway can affect the function of immunosuppressive cells, providing new ideas for immunotherapy. In this study, we summarize the activation pathway of the cGAS-STING pathway and its immunological function and elaborate on the key role of this pathway in immune escape mediated by the tumor immunosuppressive microenvironment. Finally, we summarize the mainstream immunotherapeutic approaches related to this pathway and explore ways to improve them, thereby providing guidelines for further clinical services.

## 1 Introduction

The emergence of tumor immunotherapy has significantly improved the prognosis and prolonged survival of cancer patients over the past decade (1). Current mainstream immunotherapies include immune checkpoint blockade (ICB) (2), chimeric antigen receptor T-cell (CAR-T) immunotherapy (3), and monoclonal antibody therapy (4). The goal of most immunotherapies is to enhance adaptive anti-tumor immunity. Indeed, adaptive anti-tumor immunity is highly dependent on strong innate immunity (5). Innate immunity, the first immune barrier of an organism, plays an important role in combating the invasion of foreign pathogenic microorganisms and maintaining homeostasis (6).

The cGAS-STING pathway has emerged as a critical part of the innate immune defense of the host, and its role in tumor immunity has been elucidated. Numerous studies have demonstrated that activating the cGAS-STING pathway can influence the efficacy of tumor immunotherapy. The combination of STING-related agonists significantly improves patient prognosis and reduces the occurrence of immunotherapy resistance (7, 8). Drugs targeting this pathway may become more widely available, as evidence suggests that the cGAS-STING pathway is an excellent tumor target.

Tumors continuously promote the fusion of surrounding tissues during their initiation and development, creating a microenvironment conducive to tumor growth known as the tumor microenvironment (TME) (9). Tumor-associated immune cells, including M2 macrophages (10), MDSC (11), regulatory T (Treg) cells (12), immune factors, extracellular matrix, and other components, interact with tumors to form an immunosuppressive microenvironment, mediating tumor immune escape and leading to immunotherapy failure (13). The cGAS-STING pathway can bidirectionally regulate the effects of immunosuppressive cells, and targeting the cGAS-STING pathway can influence the function of immunosuppressive cells.

In this study, we reviewed the immune function of the cGAS-STING pathway, elaborated on its key role in the immunosuppressive microenvironment-mediated immune escape of tumors, summarized the relationship with mainstream immunotherapeutic approaches, explored ways to improve these immunotherapies to further serve the clinic, and provided guidance suggestions.

## 2 cGAS-STING pathway activation and its immune function

**Figure 1:**
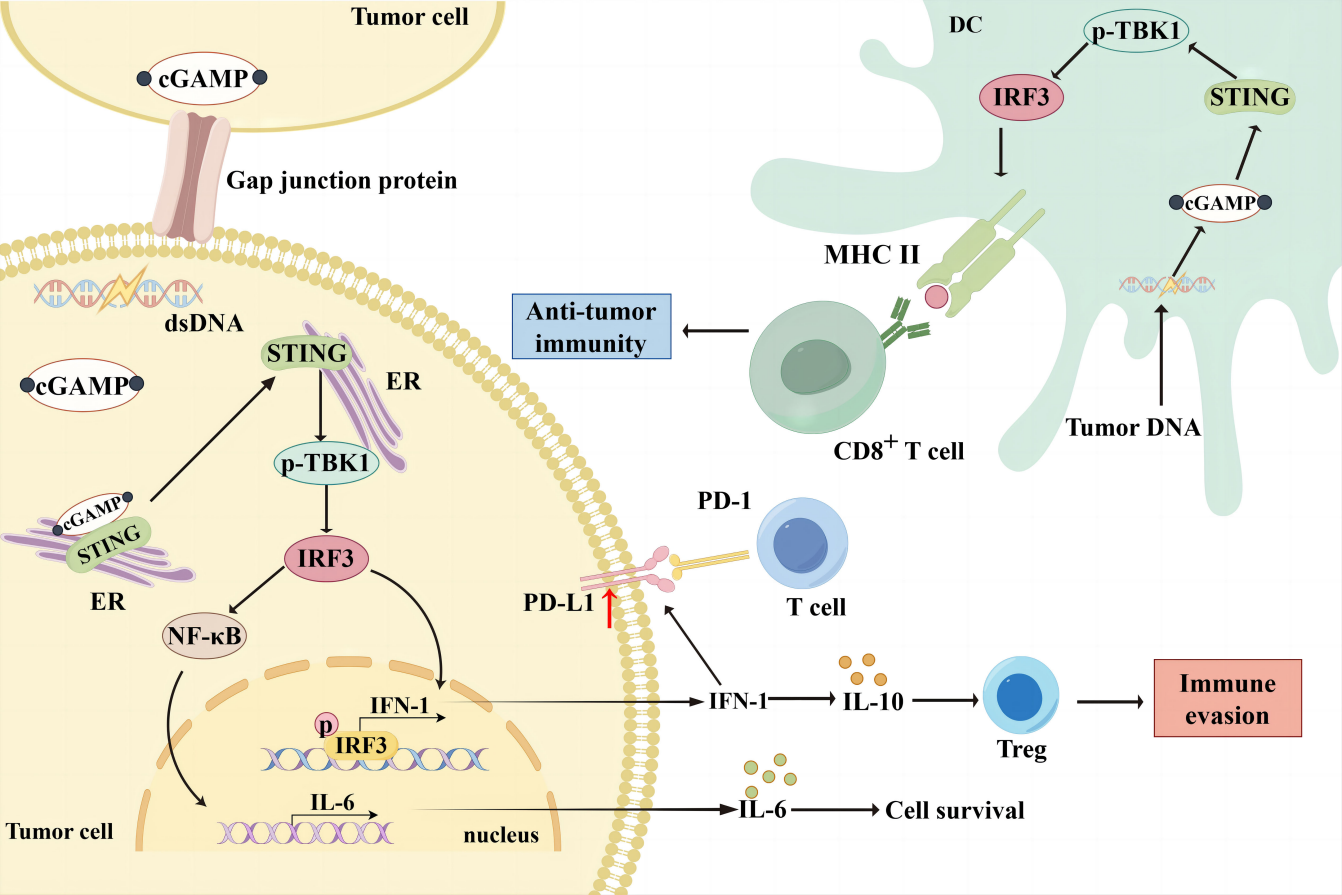
Bidirectional immunomodulation using cGAS-STING. When cGAS binds to cytosolic DNA in tumor cells, it can form 2'3'-cGAMP, which translocates to the ER as a second messenger and activates the transmembrane receptor protein STING. The 2'3'-cGAMP can also be translocated from one cell to another via cellular gap junction proteins, thereby activating the STING cascade signaling in other cells. Activated STING interacts with TBK1 and promotes TBK1 phosphorylation, thereby phosphorylating STING. Phosphorylated STING activates IRF3, which translocates to the nucleus and activates IFN-I gene transcription, thereby promoting IFN-I secretion from tumor cells. However, IFN-I upregulated PD-L1 expression in tumor cells and reduced the immune response. Additionally, IFN-I induced Treg infiltration by upregulating IL-10 expression, leading to immune escape. Moreover, STING mediates NF-κB activation and promotes IL-6 secretion, thereby promoting tumor cell survival. DCs in the TME take up broken tumor DNA, initiating the cGAS/STING/TBK1/IRF3 pathway and promoting MHC molecule expression. Subsequently, DCs can present tumor antigens to T cells and induce CD8+ T cell activation to exert anti-tumor effects.

The cGAS-STING signaling pathway is an innate immune defense pathway that has evolved to combat pathogenic microbial infections (14). It is multifunctional, and dysregulation can disrupt cellular and organismal homeostasis by triggering various abnormal innate immune responses associated with pathology (15). Increasing evidence suggests that the cGAS-STING pathway is involved in tumorigenesis, metabolism, immunomodulation, and immunosuppression and can modify the TME to participate in tumorigenesis (16). The cGAS is a cytosolic DNA sensor or receptor that binds directly to DNA and is activated in the presence of cytosolic DNA (17). The cGAS has two double-stranded DNA (dsDNA) binding sites, and upon binding to DNA, it dimerizes from its inactive to active form and undergoes a conformational change (15, 18, 19). The cGAS dimer catalyzes the formation of a phosphodiester bond between ATP and GTP, forming 2′3′-cGAMP (20). Post-translational modifications can regulate cGAS activation at the transcriptional level, with acetylation and phosphorylation affecting cGAS activation, allowing the possibility of modulating the cGAS-STING signaling pathway (19, 21, 22). The 2′3′-cGAMP is a cyclic dinucleotide that acts as a second messenger that translocates into the endoplasmic reticulum (ER) and activates the transmembrane receptor protein STING (23). Due to its conformational specificity, 2′3′-cGAMP has also been reported to transfer from one cell to another via cellular gap junction proteins to activate the STING cascade signaling in other cells (24). STING is an ER membrane-bound protein with binding sites for TANK-binding kinase 1 (TBK1) and interferon regulatory factor 3 (IRF3). Numerous inactive STING dimers exist in the ER, and many TBK1 molecules can bind to STING dimers to form an inactive STING-TBK1 complex. The 2′3′-cGAMP activates STING upon binding to STING. Activated STING interacts with TBK1 to promote autophosphorylation of the TBK1 CTT region, which is also phosphorylated by TBK1 (25–28). Phosphorylated STING binds to the positively charged region of IRF3, leading to IRF3 activation and conformational changes. The activated IRF3 dimer translocates to the nucleus and activates the transcription of type I interferon (IFN-I) and IFN-stimulated genes, promoting the cellular secretion of IFN-I (29).The most direct effect of IFN-I is to induce dendritic cell (DCs) maturation and mediate anti-tumor immunity (30). IFN-I can be divided into IFN-α and IFN-β. Both are slightly different in structure and function (31). Among other things, IFN-α not only promotes the localization of MHC-I to antigenic storage compartments within DCs, but also increases the levels of MHC-I and MHC-II at the cell membrane (32). Tumor cells can induce their own and DCs to produce IFN-β and thus participate in the immune response (33, 34). Dan et al. likened IFN-I to the bridge between the cGAS-STING pathway and CD8^+^ T cell-mediated anti-tumor immunity (5). After the uptake of tumor DNA, DCs activate the IFN pathway by activating STING and inducing tumor antigen expression via MHC in the TME. Subsequently, DCs can present tumor antigens to T cells and induce CD8^+^ T activation (35). Meanwhile, the activation of natural killer (NK) cells and fibroblasts is inextricably linked to the cGAS-STING pathway (36) (**Figure 1**).

However, as the function of the cGAS-STING pathway remains to be elucidated, there is increasing evidence that it mediates anti-tumor immunity and plays a key role in promoting malignant tumor progression. Under normal conditions, eukaryotes maintain a strict boundary between DNA and the cytoplasm to avoid autoimmunity caused by unwanted contacts (37). Genomic instability and DNA damage in tumor cells can lead to the appearance of abnormal DNA in tumor cells (38). Cancer cell proliferation causes genomic instability, usually characterized by the segregation of chromosome mismatches during mitosis. Due to segregation defects, lagging chromosomes give rise to micronuclei in a cell cycle-dependent manner (39). The micronucleus envelope ruptures readily without a stable nuclear membrane, exposing its genomic content to the cytoplasm (40, 41). In triple-negative breast cancer (TNBC), chromosomal instability causes cGAS-STING-dependent IL-6 production. Upregulated IL-6 and NF-κB prevent STAT1 and ASK-JNK-mediated cell death, leading to tumor cell survival (42). DNA damage includes endogenous DNA damage during mitosis or exogenous DNA damage induced by radiotherapeutic or chemotherapeutic agents. Deletion of the MutLa subunit MLH1 disrupts DNA repair. MutLa specifically regulates exonuclease 1 (Exo1). MutLa can specifically regulate Exo1, leading to unrestricted excision of DNA due to the altered structure of MutLa, increased formation of single-stranded DNA, release of abnormal chromosomal and nuclear DNA into the cytoplasm, and activation of the cGAS-STING signaling pathway (41). Exposure to ionizing radiation or chemotherapeutic treatments, such as platinum-based drugs, can also induce DNA double-strand breaks and activate the cGAS-STING pathway to maintain tumor cell survival (43). This may be relevant to tumor recurrence and drug resistance.

Besides the aberrant production and release of nuclear DNA, mitochondrial DNA (mtDNA) may activate the cGAS-STING pathway. In some malignant cells experiencing oxidative stress and mitochondrial dysfunction, mtDNA may also be released into the cytoplasm due to excessive oxidative stress and reactive oxygen species (ROS) or structural damage to the mitochondrial membrane, thereby mediating the cGAS-STING cascade signaling pathway (44). When the mitochondrial protein Lon is overexpressed in oral cancer, oxidized mtDNA is released into the cytoplasm and activates the cGAS-STING-IFN signaling loop, thereby inhibiting T cell activation by upregulating the expression of PD-L1 and IDO (45). Drp1 overexpression in esophageal squamous cell carcinoma can cause mitochondrial dysfunction, inducing mtDNA release to activate STING, triggering autophagy, and promoting tumor cell proliferation and migration (46). OMA1 is a metalloproteinase located in the inner mitochondrial membrane. OMA1 interacts with HSPA9 to induce mitochondrial phagocytosis in gliomas. OMA1 acts as an immune evader by increasing mtDNA release, activating the cGAS-STING pathway, and promoting PD-L1 transcription (47). Additionally, tumor cells can spontaneously take up mtDNA from the TME, promoting tumor survival by activating the cGAS-STING pathway (41, 48).

## 3 The role of the cGAS-STING pathway in tumor immune evasion

The human immune system constantly removes “non-self” factors to maintain homeostasis. The emergence of tumors indicates that tumor cells use certain pathways to evade the body’s surveillance (49). Immune escape of tumor cells has become a major obstacle in tumor immunotherapy, and eliminating immune escape may improve the prognosis of tumor patients (50). Immunoediting and immunosuppressive microenvironments are key aspects of tumor escape. The former results in the absence of tumor cell-specific antigens and low expression of MHC molecules, thereby hindering the recognition of tumor cells by T lymphocytes. This reduces the immune response of the body to the tumor via various pathways, including immunosuppressive cells and cytokines, thereby ensuring tumor cell survival (51).

The cGAS-STING pathway regulates immune escape through several mechanisms. Among tumor-associated T cells, LRRC8C-enriched T cells can mediate immune escape by transporting cGAMP and activating the STING-p53 axis to suppress T cell-dependent adaptive immunity (52). Tumors can also mediate T cell death by activating the STING-IFN pathway in T cells, which can be blocked using STING inhibitors (53). Overexpression of the mitochondrial protein Lon releases oxidized mtDNA into the cytoplasm, mediating immunosuppression by activating the IFN pathway via cGAS-STING-TBK1, upregulating PD-L1 and IDO-1 expression and inhibiting T cell activation (43). The cGAS-STING pathway effector molecule, IFN-β, can also exert immunosuppressive effects. IFN-β in IFN-I induces Tregs infiltration by upregulating IL-10 expression, leading to immune escape (54, 55). IFN-I can also induce radiation resistance by promoting the recruitment of immunosuppressive myeloid cells via the CCR2 pathway (56). Sustained IFN-I (IFN-α and IFN-β) activation can induce upregulation of PD-L1 in tumors and DCs, which in turn increases NOS2 expression, ultimately leading to failure of PD-1 immunotherapy (56, 57). Besides these mechanisms, direct DNA-mediated activation of the cGAS-STING pathway has been implicated in immune escape. DNA damage activates STING signaling, and STING-mediated activation of NF-κB enhances IL-6-mediated STAT3 expression in TNBC cells, thereby inducing tumor cell survival and immunosuppression (58). Nucleotidase ENPP1 selectively degrades extracellular cGAMP to mediate immunosuppression. cGAMP can generate immunosuppressive adenosine after degradation, thereby reducing immune cell infiltration (**Figure 1**) (59).

## 4 cGAS-STING signaling in immunosuppressive cells

### 4.1 cGAS-STING pathway and macrophages

TAMs are important immune cells in the TME that play key roles in tumor invasion, drug resistance, malignant proliferation, and metastasis. TAMs receive signals from the TME and perform various immunological functions (60). Generally, naive macrophages (M0) can be polarized into two primary subpopulations: M1 and M2. M1 macrophages induce inflammation and play an important role in eliminating pathogens, tumors, and foreign bodies. M2 macrophages are key cells in tumor development because they reduce the immune response and promote immune escape (61). Additionally, M2 macrophages can be subdivided into four subpopulations: M2a, M2b, M2c, and M2d.

The primary phenotypic markers of M1 macrophages are CD80/86^high^, MHCII^high^, TLR2, TLR4, and CCR7^high^, which generally inhibit cancer. The primary phenotypic markers of M2a macrophages are CD206^high^, CD209^high^, Dectin-1^high^, CD163^low-medium^, CD86^low^, CD14^low-medium^, and IL-1R, which promote tissue repair, tumor cell proliferation, metastasis, and invasion. The primary phenotypic markers of M2b macrophages are CD163^low^, CD86^medium^, MerTK^medium-high^, CD16, TLR1, and TLR8, which can phagocytose apoptotic cells. The primary phenotypic markers of M2d macrophages are CD163^high^, CD86^low^, and CD14^high^, which can promote angiogenesis and tumor metastasis (62). Tumor tissues can recruit and alter the phenotype of macrophages to favor M2 macrophages by remodeling the immune microenvironment (63, 64). The pro-tumorigenic role of M2 macrophages is an important factor in tumor recurrence after surgical resection (65).

**Figure 2:**
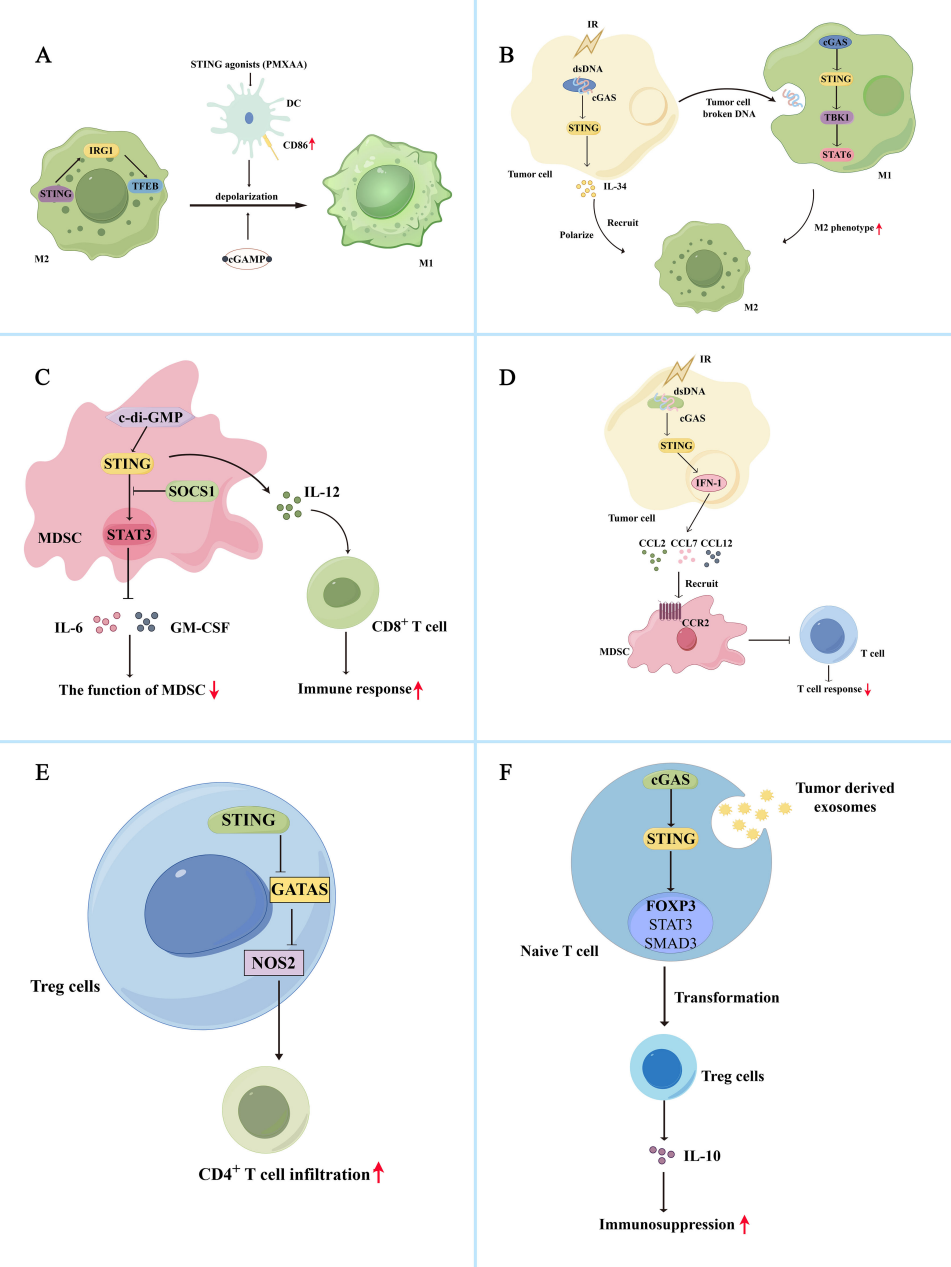
Bidirectional regulation of the cGAS-STING pathway and immune cells in the tumor immunosuppression microenvironment **(A)** The cGAS-STING pathway inhibits M2 macrophage polarization and promotes anti-tumor immunity. The STING agonist, PMXAA, promotes DC overexpression of the co-stimulatory molecule CD86, thereby reprogramming immunosuppressive M2 macrophages into immune-activating subtype M1 macrophages. Meanwhile, STING agonists, DMXAA and 2’3’-cGAMP, can reprogram M2 macrophages into M1 macrophages *in vitro*. Additionally, STING activates IRG1, promotes TFEB nuclear translocation, inhibits M2 macrophage polarity, and reduces immunosuppression. **(B)** The cGAS-STING pathway promotes M2 macrophage polarization and immunosuppression. Radiation-exposed tumor cells can produce broken DNA, activate the cGAS-STING pathway, promote IL-34 secretion, and promote M2 macrophage polarization and recruitment, thereby promoting tumor cell survival. Simultaneously, tumor cells can release degraded DNA into the TME for macrophage uptake. Macrophage uptake activates the cGAS/STING/TBK1/STAT6 signaling pathway, inducing the generation of M2 macrophages and promoting apoptosis of M1 macrophages. **(C)** Activation of the cGAS-STING pathway induces inactivation of MDSCs, reducing their immunosuppressive function. The c-di-GMP activates the cGAS-STING pathway in MDSC, converting immunosuppressed MDSC subpopulations into an IL-12-producing immunostimulatory phenotype that enhances the CD8^+^ T cell-mediated immune response. Additionally, STING signaling activates SOCS1 protein, physically interacting with STAT3, preventing its phosphorylation and dimerization, and reducing the immunosuppressive function of MDSCs by inhibiting the production of GM-CSF and IL-6. **(D)** Activation of the cGAS-STING pathway also promotes the recruitment of MDSCs, thereby exerting their immunosuppressive function. After irradiation, tumor cells activate the STING/IFN-I pathway and release the chemokines CCL2, CCL7, and CCL12 via the CCR2 pathway to recruit MDSCs. The recruited MDSCs reduce the immune response of T cells and exert immunosuppressive functions. **(E)** Activation of cGAS-STING attenuates Treg-mediated immunosuppression. In Tregs, activation of STING decreased GATA3/NOS2 expression, which is associated with immunosuppression, increased CD4^+^ T cell infiltration, and reduced immunosuppression. **(F)** Activation of the cGAS-STING pathway promoted Treg cell-mediated immunosuppression. Tumor-derived exosomes activate the cGAS-STING pathway in initial lymphocytes, activate Foxp3, STAT5, and SMAD3, and promote the conversion of initial CD4^+^ T cells into Treg cells, thereby mediating immunosuppression.

The cGAS-STING pathway and its downstream effects mediate the polarization of tumor-associated macrophages. The cGAS-STING pathway inhibits M2 macrophage polarization and promotes anti-tumor immunity. The cGAS-STING agonists promote the expression of co-stimulatory molecules in DCs and reprogram M2 macrophages with immunosuppressive functions into immuno-activated subtype M1 macrophages (66). Additionally, the STING agonists, DMXAA and 2′3′-cGAMP, can repolarize M2 bone marrow-derived macrophages to M1 macrophages *in vitro*, inducing tumor site-specific vascular disruption and reducing tumor burden in non-small cell lung cancer (NSCLC) mouse models (**Figure 2A**) (67). Worryingly, phase III clinical trials showed that DMXAA did not improve first-line efficacy in advanced NSCLC (68). However, it showed good results in mouse models (69). In colorectal cancer liver metastasis, STING can activate IRG1, promote nuclear translocation of TFEB, inhibit the polarization of M2 macrophages, and reduce the ability of macrophages to promote tumor metastasis (70). Additionally, NAMPT deficiency significantly reduced the efferocytosis activity of macrophages, increasing the STING pathway and IFN-I gene expression activity, promotes IFN-β production, and consequently reduces M2-type macrophage polarization (71). The hypoxic TME promotes the release of numerous exosomes from glioma cells and increases the expression of miR-25/93 in these hypoxia-derived exosomes. Macrophages take up this group of hypoxia-derived exosomes and miR-25/93, inhibiting the cGAS-STING pathway, reducing IFN-β secretion, and downregulating M1 polarization-related gene expression (CXCL9 and CXCL10), thereby reducing anti-tumor immunity (72). MARCO is a macrophage receptor with a collagen structure, and its high expression can enhance the immunosuppressive function of macrophages (73–75). It is also negatively correlated with the prognosis of hepatocellular carcinoma. In contrast, MARCO^+^ TAM has strong phagocytic ability and can rapidly remove dying tumor cells from the TME, minimizing the accumulation of tumor-derived cGAMP and ATP. The lack of extracellular ATP inhibits P2X7R-mediated cGAMP transport on TAM surfaces. It also inhibits activation of the cGAS-STING pathway, reducing IFN-I secretion by macrophages and immunosuppression (76).

In addition, the cGAS-STING signaling pathway is involved in forming M2 macrophages. Tumor cells may shed broken DNA or particles into the TME for macrophage uptake. After uptake, macrophages can activate the cGAS/STING/TBK1/STAT6 pathway, inducing the formation of an M2 phenotype and promoting apoptosis of M1 macrophages (77). In esophageal squamous cell carcinoma, irradiation of tumor cells can activate the cGAS-STING pathway and promote IL-34 secretion, thereby promoting the polarization and recruitment of M2 macrophages and tumor cell survival (**Figure 2B**) (78). Activation of the cGAS-STING pathway in macrophages induces IFN synthesis and secretion, leading to overexpression of BST2 in macrophages. BST2^+^ macrophages secrete CXCL7 via the ERK pathway and bind to CXCR2, activating the AKT/mTOR pathway and promoting CD8^+^ T cell exhaustion, thereby contributing to the poor prognosis of pancreatic ductal adenocarcinoma (79). Additionally, Zhang et al. demonstrated that circASPH promotes M2 macrophage polarization by stabilizing the IGF2BP2 protein and increasing the stability of m6A-modified STING mRNA (10). In lung adenocarcinoma, IFITM1 upregulates the expression of IL-1α/1β, VEGFA, and IL-6 by activating the STING-TBK1-IRF3 pathway, promoting monocyte recruitment, and M2 macrophage polarization, resulting in immune suppression (80).

### 4.2 cGAS-STING pathway and MDSC

MDSCs are a heterogeneous population of immature bone marrow cells that induce T cell inactivation and mediate immunosuppressive responses. MDSCs are rarely found in the blood of normal individuals but appear when the body is exposed to severe immune disorders, pathological injury, inflammatory storms, and others (81, 82). MDSCs consist of two primary subpopulations: monocyte-like MDSCs (M-MDSCs) versus granulocyte-like MDSCs (PMN-MDSCs or G-MDSCs). The molecular markers of M-MDSCs are CD11b^+^, CD33^high^, HLA-DR^−^, CD14^+^, and CD15^−^. The molecular markers of PMN-MDSCs and G-MDSCs are CD11b^+^, CD33^medium^, HLA-DR^−^, CD14^−^, CD15^+^, and CD66b^+^ (83). M-MDSCs are predominantly mediated by the high TGF-β, arginase (Arg1), and iNOS expression levels to mediate the non-specific inactivation of T cells. PMN-MDSCs primarily produce high ROS levels and mediate immunosuppression through direct cellular contact with T cells, reducing antigen-specific T cell responses without affecting the response to non-specific stimuli (84). Due to the highly heterogeneous nature of MDSCs and the complexity of their function, the mechanism of the role of MDSCs in tumor immunosuppression is currently unknown and requires further investigation.

Studies have demonstrated that activating the cGAS-STING pathway can induce inactivation of MDSCs, thereby reducing their immunosuppressive function. The cGAMP, the initiator of the STING pathway, can activate CD8^+^ T cells to produce IFN-γ and inhibit ROS and nitric oxide (NO) production in MDSCs, thereby attenuating MDSC-mediated immunosuppression. And the number of MDSCs, PMN-MDSCs and M-MDSCs in tumor tissue was reduced after treatment with cGAMP (85). The c-di-GMP, a compound like cGAMP, can act as an activator of STING proteins, activate the cGAS-STING pathway in MDSC, and convert a subpopulation of immunosuppressed MDSCs to an IL-12-producing immunostimulatory phenotype, thereby improving the CD8^+^ T cell-mediated immune response (**Figure 2C**) (86). In addition, STING signaling can activate SOCS1 protein, which can physically interact with STAT3 via its SH2 structural domain to prevent the phosphorylation and dimerization of STAT3 and reduce the immunosuppressive function of MDSCs by inhibiting GM-CSF and IL-6 production (87). Besides inactivating MDSCs, activation of the cGAS-STING pathway promotes the recruitment of MDSCs to exert their immunosuppressive function. After irradiation, tumor cells activate the STING/IFN-β signaling pathway to release chemokines, including CCL2, CCL7, and CCL12, via the CCR2 pathway to recruit M-MDSC. The recruited M-MDSC reduced the T cell immune response to exerting their immunosuppressive function (**Figure 2D**) (56). CCR2 antibodies can reduce radiation-induced recruitment of MDSCs and attenuate their immunosuppressive function. IFN-β, a downstream signal of the cGAS-STING signaling pathway, can also stimulate tumor cells to produce CCL2 and CCL7 and affect the recruitment of M-MDSC (88). Besides the STING/IFN-I pathway, STING-mediated activation of the NK-κB pathway is closely linked to MDSC recruitment. For example, galectin-1 maintains NF-κB activation in tumor cells by enhancing STING protein stability, thereby promoting CXCL2-mediated PMN-MDSC recruitment (89).

### 4.3 cGAS-STING pathway and Treg cells

Tregs are involved in forming the immunosuppressive microenvironment and immune tolerance. They are characterized by CD4^+^ Foxp3^+^ CD25^+^ CTLA-4^+^ as their major molecular feature (90). Foxp3 regulates CTLA-4 expression in Treg cells, which can bind to CD80/CD86 on APCs, affecting their messaging and inhibiting T-lymphocyte activity. Anti-CTLA-4 monoclonal antibodies with ADCC activity can reduce Treg cells in the TME to attenuate tumor recurrence (91, 92). Additionally, Treg cells regulate immune function by downregulating co-stimulatory signals, depleting IL-2, releasing immunosuppressive cytokines IL-10 and IL-35, and producing immunosuppressive metabolites (93).

Thus, the cGAS-STING-IFN pathway may influence the immunosuppressive function of Tregs. Activation of cGAS-STING attenuates Treg-mediated immunosuppression. Sallets et al. discovered that STING activation reduced the proportion of tumor-infiltrating CD4^+^ Foxp3^+^ Treg cells (94). Domvri et al. found that decreased STING elevated GATA3/NOS2 expression associated with immunosuppression in Tregs, reduced CD4^+^ T cell infiltration, and increased the risk of subsequent lung metastasis (**Figure 2E**) (95). In a mouse model of melanoma, injection of cGAMP packaged in non-infectious enveloped virus-like particles preferentially activated STING in DCs, differentiating circulating tumor-specific T cells, thereby reducing Tregs and exerting anti-tumor effects (96). The cGAS-STING pathway bi-directionally regulates the effects of Tregs, and its activation promotes Treg cell-mediated immunosuppression. The STING downstream signal IFN-I enhances immunosuppressive effects by driving tumor-associated infiltrating Tregs to produce IL-10 (97). Tumor-derived exosomes activate the cGAS-STING pathway in naive lymphocytes, activating Foxp3, STAT5, and SMAD3 to promote the transformation of naive CD4^+^ T cells into Treg cells, thereby mediating immunosuppression (**Figure 2F**) (98). The cGAS-STING pathway also modulates mitochondrial lipid metabolism in Tregs, thereby enhancing Treg cell function. FABP5 is a lipid-binding protein that reduces the β-oxidation rate and accumulates lipid droplets in monocytes. Monocytes secrete more IL-10 with the help of FABP5, and elevated IL-10 levels promote PD-L1 expression in Tregs by activating the JNK-STAT3 pathway. PD-L1 expression mediates immunosuppression (99). However, in Tregs, FABP5 inhibition triggers mtDNA release and activation of the cGAS-STING-IFN-I pathway, inducing IL-10 production and promoting the immunosuppressive activity of Tregs (100). FABP5 plays different roles in different cells. However, evidence suggests that FABP5 is associated with activation of Tregs and the cGAS-STING pathway. Based on these different perspectives, it is important to comprehensively understand the cGAS-STING pathway involved in forming the immunosuppressive microenvironment.

## 5 cGAS-STING pathway and immunotherapy

### 5.1 cGAS-STING pathway and immune checkpoint inhibitors

Immunotherapy with PD-1/PD-L1 immune checkpoint inhibitors is an effective cancer treatment (101). The cGAS-STING pathway-related agonists can synergistically interact with PD-L1 inhibitors to exert anti-tumor immune functions. In a phase Ib clinical trial (NCT03172936) of advanced/metastatic solid tumors or lymphomas, the combination of the STING agonist MIW815 (ADU-S100) and spartalizumab (PDR001), a monoclonal antibody directed against PD-1, significantly reduced patient discomfort and improved patient prognosis (**Table 1**) (102). In another Phase I clinical trial (NCT03010176), the combination of a STING agonist (MK-1454) and PD-1 antibody (pembrolizumab) prolonged survival in patients with advanced solid tumors, illustrating its potential for clinical use (103). The synergistic effect of STING agonist and PD-1 ICB enhances the response of high-grade plasma ovarian cancer to carboplatin-based chemotherapy in mice and promotes the killing effect of carboplatin on cancer cells (104). In a mouse model of cervical cancer, the STING agonist MSA-2, in combination with a PD-1 monoclonal antibody, significantly prolonged the survival cycle of mice, and MSA-2 administration remodeled the TME and exerted anti-tumor activity in mice (105). In a mouse model of breast cancer, STING agonists promoted the activation of the STING/TBK1/IRF3/STAT1 pathway, releasing IFN-β, thereby enhancing the efficacy of the PD-L1 monoclonal antibody. Simultaneously, STING agonists, in combination with the PD-L1 monoclonal antibody, increased the number of CD8^+^ cytotoxic T cells and decreased the number of FOXP3^+^ Treg cells, further prolonging the survival of mice (106). In another related study, combining the oral STING agonist MSA-2 and the anti-TGF-β/PD-L1 bispecific antibody YM101 was a novel immune cocktail therapy for treating unwanted tumors (107). These data suggest that the synergistic application of STING agonist PD-1/PD-L1 monoclonal antibodies may be a key factor in improving patient prognosis.

**Table 1:** Special features of the three immunotherapies.

	Types of tumors	Veterinary drug	Target or mechanism of action	Genus	Reference
The cGAS-STING pathway and immune checkpoint inhibitors	Advanced/metastatic solid tumors or lymphomas	the STING agonist MIW815 (ADU-S100) and spar talizumab (PDR001)	PD-1	Human [Phase Ib clinical trial (NCT03172936)]	(102)
	Advanced solid tumor	STING agonist MK-1454 and PD-1 antibody pembrolizumab		Human [Phase I clinical trial (NCT03010176)]	(103)
	High-grade plasma ovarian cancer	STING agonist 2′3′-c-di-AM and anti-mouse PD-1 antibody (clone RMP1-14)		Mouse	(104)
	Cervical cancer	STING agonist MSA-2 and PD-1 antibody		Mouse	(105)
	Breast cancer	STING agonist c-di-GMP and atezolizumab	PD-L1	Mouse	(106)
	Melanoma, colorectal cancer, breast cancer, liver cancer	oral STING agonist MSA-2 and anti-TGF-β/PD-L1 bispecific antibody YM101	TGF-β/PD-L1	Mouse	(107)
The cGAS-STING pathway and CAR-T therapy	Breast cancer	STING agonist DMXAA or cGAMP	CAR-T generated by Th/Tc17 cells	Mouse	(104)
	Kidney cancer	PARP inhibitors (PARPis)	cGAS-STING signaling pathway	Mouse	(123)
	Prostate cancer, pancreatic cancer, lymphoma, breast cancer	PD-L1 inhibitor atezolizumab and CAR-T	cGAS-STING signaling pathway	Mouse	(128)
The cGAS-STING pathway and monoclonal antibody immunotherapy	Head and neck tumors	STING agonist 2′, 3′-GAMP and cetuximab	NK cell activation and DC maturation	Mouse	(133)
	NSCLC	Osimertinib with anti-HER3 monoclonal antibody	cGAS-STING signaling pathway	Mouse	(132)
	NSCLC	cetuximab plus avelumab	NK cell-driven activation of ADCC and cGAS-STING signaling pathways	Mouse	(134)
	Lymphomas	STING agonist [human (2’2’-, 2’3’- and 3’3’- cGAMP) and murine (DMXAA)] and anti-CD20 mAb	reverse the inhibitory effect of lymphoma on macrophage FcγR expression	Mouse	(133)
	Advanced/ recurrent solid tumors that express HER2	a STING ADC drug XMT-2056	Erbb2 tyrosine kinase receptor modulator. STING stimulator	Human [Phase 1 Clinical (NCT05514717)]	(141)

In two trials of platinum-based drugs for treating tumors, either carboplatinum or teniposide activated the cGAS-STING pathway and its downstream classical STING/TBK1/IRF3 pathway, as well as atypical STING-NF-κB signaling under certain conditions, enhancing the anti-tumor effect of PD-1 monoclonal antibodies in tumor immunity (108, 109). The mechanism of action of these chemotherapeutic agents is to induce DNA fragmentation in tumor cells, and these broken DNA molecules activate the cGAS-STING pathway in different ways. Similarly, anti-cancer drugs targeting ADP-ribose polymerase inhibitor (PARPi) can activate the cGAS-STING pathway by inducing cytosolic micronuclei, promoting the secretion of chemokines, such as CCL5, through IFN-γ-induced PD-L1 expression on the tumor cell surface, and the combination of PARP and PD-L1 monoclonal antibody significantly improves the prognosis of patients (5). When tumor-infiltrating T cells were stimulated with a combination of anti-CD3 and anti-PD-1 monoclonal antibodies, the STING/IFN-γ pathway was induced and activated in lung adenocarcinomas, increasing the IFN-β and CCL5 expression, and an active IFN-γ pathway is a common feature of tumors responding to PD-1/PD-L1 blockade therapy (110). However, other studies have reported that prolonged IFN-β stimulation induces NOS2 expression and promotes Treg cell generation, ultimately leading to the failure of PD-1 immunotherapy (56, 57). Certain intestinal flora also affect the therapeutic effects of PD-1/PD-L1 antibodies via STING-related pathways, such as *Listeria monocytogenes* strain GG (LGG), inducing cGAS/STING-dependent IFN-β production in DCs and enhancing the response to PD-1 ICB therapy (111).

The STING-IFN-I pathway activity and antigen-presenting capacity were significantly reduced in aged mice with TNBC. Age-related immune dysfunction limits the efficacy of ICB in aged mice with TNBC. Induction of innate immunity with STING agonists can restore the response to ICB in aged mice (112). Other studies have suggested that the integrity of the STING-related pathway is critical to the outcome of immunotherapy with CTLA-4. A study revealed that systemic treatment with STING agonists in combination with α-PD-1 and α-CTLA-4 antibodies disappeared abdominal tumors in approximately 71% of mice (113).

### 5.2 The cGAS-STING pathway and CAR-T therapy

Chimeric antigen receptor (CAR)-T cells are engineered cells that express CARs against specific tumor antigens (114). CAR-T cells can be activated in an MHC-independent manner and can directly kill tumor cells (115). CAR-T therapy has demonstrated great therapeutic promise for hematological diseases, including childhood acute lymphoblastic leukemia and lymphoma (116). Certain barriers to the effectiveness of CAR-T therapy in solid tumors must be addressed in further clinical trials. These barriers include the heterogeneity of T cells, difficulties in transporting them from the blood to the tumor site, immunosuppression of the TME, and exhaustion of CAR-T cells (117).

Activation of the cGAS-STING pathway is inextricably linked to CAR-T therapy; therefore, it appears to be a good target for improving the prognosis of CAR-T therapy. The STING agonists DMXAA or cGAMP promote the secretion of chemokines, including CCL2 and G-CSF, and reduce the suppressive effects of the immune microenvironment. It also promotes the migration and survival of CAR-T cells generated by Th/Tc17 cells, which benefits CAR-T cell therapy (118). The expression of the cGAS-STING cascade response in the peripheral blood CD8^+^ T cells of cancer patients was significantly impaired, which may also be related to the poor prognosis of patients. The cGAS-STING can also maintain CD8^+^ T cell stemness by regulating TCF1 expression (119). DNA damage and repair mechanisms can significantly improve the efficacy of CAR-T therapy (120). Flap structure-specific endonuclease 1 (FEN1) is highly expressed in various cancer cells and plays an important role in DNA replication and repair. A low dose of the FEN1 inhibitor SC13 increases dsDNA in the cytoplasm. Cytosolic dsDNA can activate the cyclic GMP-AMP synthase stimulator of the IFN gene signaling pathway, increase chemokine secretion, promote CAR-T cell infiltration, and enhance anti-tumor immunity (121). The PARPi are a class of cancer therapeutic agents that target PARPs (122). The PARPi stimulates chemokine secretion and facilitates CAR-T cell recruitment into the TME via the cGAS-STING pathway, thereby facilitating the efficacy of CAR-T cell therapies (123). The IFN secretion mediated by the cGAS STING pathway may also affect the prognosis of patients undergoing CAR-T treatment. The intrinsic sensitivity of IFN-γ to the pro-apoptotic effects of tumors is an important determinant of the anti-tumor activity of CD4^+^ CAR-T cells (124). IFN-γ has been demonstrated to overcome the effects of PD-L1/PD-1 inhibition on CAR-T cell therapy by upregulating ICAM-1 in tumor cells (125). oHSV1-infected glioblastomas release IFN-γ to enhance CD70-specific CAR-T therapy (126). However, CAR-T cells produce IFN-γ through the cGAS-STING pathway. IFN-γ produced by CAR-T cells enhances endogenous T and NK cell activity and is required to maintain CAR-T cytotoxicity, promote host IL-12 production, and support the host CAR-T immune response (127). CD163^+^ M2 macrophages are involved in generating an immunosuppressive microenvironment that can express PD-L1 molecules to inhibit CAR-T therapy gains, whereas PD-L1 blockade combined with CAR-T cells can lead to the loss of CD163^+^ M2 macrophages via IFN-γ signaling, improving the anti-tumor activity of CAR-T cells (128).

### 5.3 The cGAS-STING pathway and monoclonal antibody immunotherapy

Monoclonal antibody immunotherapy recognizes and destroys cancer cells by activating the patient’s immune system, thus enabling it to recognize and destroy cancer cells (129). Immune cells cannot properly receive signals to kill tumor cells because they can evade the immune system through multiple pathways. Monoclonal antibody immunotherapy promotes proper recognition and killing of tumor cells by immune cells using synthetic targeted monoclonal antibodies (130). Monoclonal antibody immunotherapy is becoming increasingly mature, and several monoclonal antibodies have been marketed and used clinically (130).

Agonists of the cGAS-STING pathway can act synergistically with monoclonal antibodies to enhance their efficacy. In head and neck tumors, STING activation enhances cetuximab-mediated NK cell activation and DC maturation, facilitating tumor-killing (131). The anti-tumor activity of monoclonal antibodies may also be linked to the cGAS-STING pathway. When osimertinib (EGFR target mutant inhibitor) was combined with an anti-HER3 monoclonal antibody to treat lung cancer, it promoted IRE1α-dependent upregulation of HER3 and activated cGAS in cancer cells to produce cGAMP, which was later transferred to macrophages and activated the cGAS-STING pathway in macrophages, thereby promoting macrophage Fc receptor-dependent tumor elimination (132). Another study exhibited that STING effectively reversed the inhibitory effect of lymphoma on macrophage FcγR expression, thereby enhancing the killing effect of CD20 monoclonal antibody on lymphoma (133). When cetuximab (an EGFR target inhibitor) was combined with avelumab (a PD-L1 target inhibitor) to treat NSCLC, the anti-cancer mechanism of these two antibodies partially depended on the activation of the ADCC and cGAS-STING pathways driven by NK cells (134).

Although monoclonal antibodies have good immunotherapeutic prospects, drug resistance still exists, and resistance to monoclonal antibodies may be associated with the cGAS-STING pathway. Trastuzumab is a key drug for treating HER2^+^ breast cancer (BC) (135). The IFI16-dependent STING signaling pathway is an important determinant of trastuzumab resistance in HER2^+^ BC. IFI16 is downregulated in HER2^+^ BC cells via synergistic histone modification by EZH2 and histone deacetylase, inducing STING/CXCL10/11 immune signaling defects associated with HER2 monotherapy and HER2 treatment resistance (136, 137).

## 6 Conclusion and future perspectives

Based on these conclusions, activating the cGAS-STING pathway is bidirectional in tumor promotion and inhibition. Its biological function may depend on the following aspects: STING-responsive target cells, immune microenvironment in which the tumor cells reside, intensity and duration of STING stimulation, tumor stage, and individual physical factors (16). These factors play critical and independent roles in the efficacy of the cGAS-STING pathway. The cGAS-STING is present in immune and tumor cells. Different cell types exhibit different biological activities. In tumor cells, STING regulates the expression of inhibitory immune molecules, including PD-L1, CCR2, IDO, and others, and evades T-cell killing, which is conducive to tumor immune escape. The simultaneous application of inhibitors of suppressor immune molecules can ameliorate the negative effects of STING agonists. Therefore, DC can promote tumor cell killing by activating the cGAS-STING pathway to secrete IFN-I. The immune microenvironment in which tumor cells live remains complex and uncharacterized. We summarized two different immune outcomes of cGAS-STING pathway activation in M2 macrophages, MDSC, and Tregs. This suggests that when using cGAS-STING pathway agonists to treat cancer, attention should be paid to the immunosuppressive microenvironment in which tumor cells live and to test for changes in immune function before and after using agonists. This may also explain the poor therapeutic efficacy of STING agonists. The duration of the STING action is also important. It is currently believed that acute and moderate STING stimulation facilitates tumor suppression, and prolonged or high-intensity STING stimulation causes immunosuppression and poor outcomes (138). For instance, chronic exposure to 7,12-dimethylbenz (a) anthracene promotes tumor cell growth in a STING dose-dependent manner (139). Besides, the tumor stage influences the efficacy of STING agonists. Activating the cGAS-STING pathway may be an effective therapeutic strategy for early and chromosomally stable tumors. However, if the tumor has already begun using STING to drive malignant progression, over-activation of STING may inadvertently worsen clinical outcomes. In advanced or metastatic tumors, STING-mediated immune function may allow aggressive tumor cells to survive (8).

Based on these factors, it is important to personalize STING-targeted therapies for different types of patients. When using STING agonists in a clinical setting, physicians must selectively activate STING signaling by carefully selecting patients and comprehensively assessing their physiology, clarifying their tumor stage, determining their CIN status, and evaluating their therapeutic window to determine which patients will benefit from drug treatment. In the use of STING agonists, constant attention should be paid to the immunotoxic effects of drug therapy, such as infectious complications of certain microorganisms, autoimmune diseases and hypersensitivity reactions. STING agonists with low immunosuppression, low immunostimulation, low likelihood of inducing hypersensitivity reactions and autoimmune diseases are what we would like to see (140). Attention should also be paid to the immunosuppressive microenvironment where tumor cells reside, although this is currently difficult to determine. Radiotherapy, targeted therapy, and immunotherapy in combination with STING agonists to treat tumors may, to some extent, circumvent the negative effects of STING activation, offering a broad research perspective. Based on the concept that different doses and durations of action of STING agonists may lead to different outcomes, it is recommended that treatment regimens be designed around acute and moderate-intensity STING agonists; however, this must be proven in further clinical trials. We aimed to identify STING activators with a low toxicity profile, high specificity, few side effects, low resistance, and long duration of action in the market and the clinic. Future STING agonists are expected to induce anti-tumor immunity in a more targeted manner, inhibit tumor cell growth and immune escape, modify the tumor microenvironment, reduce tumor microenvironmental immunosuppression as far as possible, and avoid the malignant biological behavior induced by STING activation. We aimed to alleviate pain in tumor patients and achieve greater benefits in treating tumor patients using STING agonists combined with radiotherapy, chemotherapy, immunotherapy, and other therapies.
